# Soundscape and subjective factors affecting residents’ evaluation of aircraft noise in the communities under flight routes

**DOI:** 10.3389/fpsyg.2023.1197820

**Published:** 2023-07-03

**Authors:** Fei Qu, Zhuoming Li, Tongtong Zhang, Wenjun Huang

**Affiliations:** ^1^School of Architecture and Urban Planning, Shenzhen University, Shenzhen, China; ^2^Shenzhen Key Laboratory of Architecture for Health and Well-being (in preparation), Shenzhen, China; ^3^Shenzhen Key Laboratory of Built Environment Optimization Design, Shenzhen, China

**Keywords:** aircraft noise, soundscape, health, moderating effect, non-acoustic factors

## Abstract

**Introduction:**

Aircraft noise is one of the most significant sources of environmental pollution in large cities. During the COVID-19 pandemic, strict lockdown in community might increase residents’ discomfort with the noise, which could disrupt public activities and reduce subjective well-being. Most of the existing studies considered aircraft noise as a single sound source, which have ignored the influence of other sounds in the community. This paper applied field survey to identify the soundscape and non-acoustic factors related to aircraft noise evaluation.

**Methods:**

Paper questionnaires were delivered to select residents of three sample residential areas near Shenzhen Bao’an International Airport to investigate residents’ general health, evaluation of aircraft noise, community activities, and attitudinal factors. The relationship between respondent’s noise evaluations and subjective factors were investigated through statistical analyses controlling for measured aircraft noise levels and the existence of soundscape facilities.

**Results:**

The results indicated that the negative effects of aircraft noise were enhanced during the lock down, especially for frequent space users and those residents in poor health status. Under conditions of similar levels of aircraft noise exposure, communities with more birdsong and fountain sounds had lower proportion of highly annoyed respondents and higher level of soundscape ratings. This paper further indicated that personal factors including fear of air travel, noise sensitivity, and the frequency of outdoor activity had increased the level of annoyance to aircraft noise, while higher degree of annoyance to aircraft noise was associated with poor health status.

**Discussion:**

The findings implied the moderating effects of subjective factors and the restorative effects of natural sounds, which could inform aircraft noise control and community consultation strategies by protecting vulnerable populations and creating community soundscape facilities. Future research might conduct a pre- and post-experiment to estimate the potential causal impact of the soundscape intervention.

## 1. Introduction

Community public space is an important place for residents’ daily activities and physical exercise. During the COVID-19 pandemic, strict lockdown in community placed higher demands on environmental comfort of the community public space. In some large cities, aircraft noise is one of the most significant sources of environmental pollution, especially when the road and industry noise was in a quiet state. In the past decades, studies worldwide have shown that aircraft noise can cause community annoyance and disrupt sleep (Basner et al., 2017). Long-term exposure to aircraft noise can influence psychosocial health such as hypertension, depression and stress (Faiyetole and Sivowaku, 2021; Kim et al., 2022). In China, the populations influenced by aircraft noise have been growing steadily with the aircraft expansion, resulting in more complaints and negative health impact in the under-route residential areas (Xie et al., 2014).

Examining the social and psychological triggers of resident’s noise evaluation is key to understanding the health effects of aircraft noise. Existing socio-acoustic studies have mostly considered aircraft noise as a single sound source, focusing on the subjective group response to the noise shown as dose-response curves, which have ignored the influence of other sounds in the community (Marquis-Favre et al., 2021) and non-acoustic factors. Previous experimental study has found that the modulated noise samples superimposed with birdsong and small fountain sound raised pleasure emotions and decreased stress (Qu and Xie, 2022). Such soundscape effect is worth further validation in real communities, by comparing occupants’ evaluations of aircraft noise and overall sound environments under the flight routes.

Previous studies have presented a number of possible health problems associated with aircraft noise, but have given insufficient consideration to community soundscape and non-acoustic factors. Therefore, this study used field survey to identify the factors of aircraft noise, community soundscape, and subjective attitude that influence resident’s evaluation of aircraft noise. Questionnaire study was carried out in three under-route communities to examine the effects of aircraft noise on resident’s evaluation of overall soundscape, noise annoyance and health, controlling for community and personal factors. The results can inform appropriate strategies for the reduction of aircraft noise impact that incorporate community soundscape effect and residents’ living habits.

## 2. Materials and methods

The field survey at under-route communities investigated the relationship between exposure to aircraft noise and human well-being, with a purpose of investigating the moderating effects of soundscape, community and personal factors. Paper questionnaires were delivered to select residents of three sample residential areas under the flight route near Shenzhen Bao’an International Airport. A-weighted sound pressure levels (SPLs) of aircraft noise were measured at the most frequently used open space of each community. The relationships between SPLs, respondent’s noise evaluations and health were investigated through quantitative analysis of the questionnaire data. The moderating effects of soundscape and subjective factors were examined.

### 2.1. Study sites and sample

**FIGURE 1:**
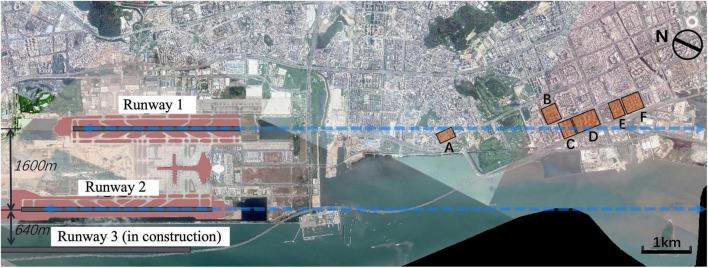
Airport configuration and selected residential areas under the flight route.

**FIGURE 2:**
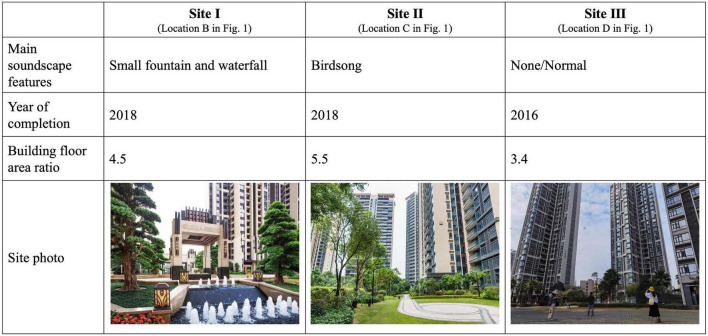
Characteristics of the study sites.

According to the flight route map and the prevailing wind condition in Shenzhen, the study first selected 6–8 representative residential areas within 10 km along the extended line of the runway (Figure 1). The selected residential areas were located under the flight routes to the east, where a large number of urban high-rise residences were built, with a high number of noise complaints from local residents. Noise measurements were carried out at selected residential areas, found that the aircraft noise exposure all exceeded the noise sensitive area limit of 57dB. Further selection was conducted in order to cover the representative soundscape and make the sample more concentrated. Three communities were finally selected as sites for questionnaire study (Figure 2). The main public space of Site I had specific soundscape featured by small fountains and waterfalls; Site II was accompanied by birdsong soundscape due to lush vegetation; whilst Site III can be used as a control group. The study calculated the sample size according to the main variables and finally randomly selected 70 adults as the sample to answer the paper questionnaires in each community. Finally, the questionnaire response rate was 91.4%, with an effective response rate of 89.0%.

### 2.2. Questionnaire design

The questionnaire collected residents’ self-assessments on wellbeing, general health and the assessment on acoustic environment including aircraft noise, as well as socioeconomic factors such as personal attitudes, activities and living conditions. The question design of specific variables mainly referred to established and widely used questions and scales. Firstly, respondents were asked to self-assess their general health on a 5-point scale from poor to excellent. Sleep disturbance in this survey was measured without making reference to noise. The question had a number of items on sleep disturbance, including “hard to fall asleep,” “sleepless deeply,” “lie awake for a while” and so on. Self-reported annoyance to aircraft noise was examined using both a verbal 5-point scale and a numerical 0–10 scale, according to the standardized ISO questions for assessment of noise annoyance (ISO, 2003). Noise sensitivity was extracted from a short form of Weinstein’s standard questionnaire (Weinstein, 1978; Zhong et al., 2018), which collected the residents’ adaptability to noise on a 6-levels numerical score. The attitude variables toward sound sources contained the frequency of air travel and the fear of flight. The moderator variables related to community contained the years of living in the community and the frequency of using public spaces. The full questionnaire can be found in Supplementary material.

### 2.3. Noise measurements

The adopted noise indicator is a globally recognized day-night equivalent A-weighted sound pressure level (*L*_*dn*_), which was obtained by measuring average noise exposure at each flyover event. The noise exposure that resident received was obtained from field measurements in main public spaces under the flight route. HEAD Acoustics’ SQobold was used for noise recording, which take into account the binaural receiving and body reflections to better record the perception of noise during aircraft overflight. To minimize the interference of background noise, the time of noise measurement was chosen at 6:00 am, 1:30 pm and 11:00 pm. The researcher was wearing the headset of SQobold with an embedded microphone and standing in the centre of the public space for recording. The daytime and nighttime noise levels were then calculated and summed separately based on the day and nighttime average flight volumes.

### 2.4. Statistical analysis

IBM SPSS 25.0 was used to create the database for the SPLs and questionnaire responses. Descriptive statistics were provided for the evaluation of aircraft noise and overall soundscape in the community. Ordinary least squared regression was applied to analyze the effects of noise and subjective covariates on self-reported annoyance. Binary logistic regression was applied to analyze the effects of noise annoyance on general health, where self-reported verbal general health was dichotomized, with fair and poor classified at “poor health status.” Odds ratios (ORs) were reported for each variable with 95% confidence intervals (CIs), with *p*-value below 0.05 considered statistically significant. The Nagelkerke psudo-R^2^ was applied as a measure of explained variance.

## 3. Results

### 3.1. Descriptive statistics of respondents and their evaluations on aircraft noise

The study received 203 responses to the questionnaire, of which 186 were valid. The questions related to the evaluations of aircraft noise have accepted reliability (Cronbach’s alpha = 0.747) and good construct validity (KMO = 0.798, Bartlett’s *p* < 0.001). The mean age in the study population was 46 (SD = 15.5), and 46% were male. The characteristics of the respondents were similar across three residential areas. No statistically significant differences were found in age, gender, education, household income, or general health status across three sites.

Overall, 97% of the respondents (*n* = 181) noticed the aircraft noise when using public spaces and 88% of the respondents (*n* = 164) were being annoyed. The proportion of highly annoyed respondents (HA%) reached 26% (*n* = 48).

The three sites had similar aircraft noise exposure, with *L*_*dn*_ of 67.5, 65.8, and 63.4 dB respectively. However, Site II and III with highly recognizable natural sounds were lower in highly annoyed ratings (HA%) than Site I (24%, 17% vs. 37%). The proportion of highly annoyed respondents was significantly different across three sites (χ^2^(2) = 6.26, *p* = 0.044).

Half of the respondents considered their level of annoyance with aircraft noise to have deepened during the COVID-19 pandemic, while 23% considered their degrees to have reduced, 20% reported no change. Pearson’s Chi-square study showed that those respondents indicating increased annoyance during the pandemic were more likely to use public spaces frequently (χ^2^(1) = 11.91, *p* = 0.001) and in poor health status (χ^2^(1) = 36.49, *p* < 0.001).

Respondent’s self-assessed happiness and sleep status were not associated to aircraft noise annoyance. The study found that respondent’s annoyance levels to aircraft noise were related to subjective factors including soundscape evaluation, community space using, psychological and personal factors.

### 3.2. Evaluations on the community soundscape

**FIGURE 3:**
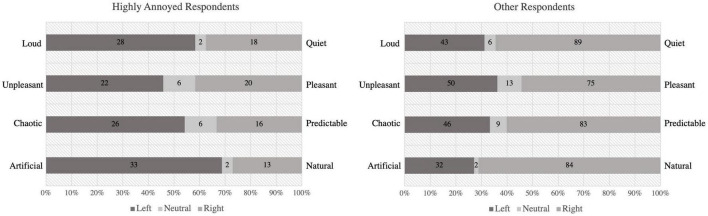
Evaluations of the sound environment between highly annoyed (*n* = 48) and other respondents (*n* = 138) with number of respondents in each category.

Respondents evaluated the sound environment of the public space using eight semantic adjectives rated on unipolar scales. Overall, respondents gave positive ratings of the community’s soundscape. However, the evaluations were different between highly annoyed and the rest of the respondents (Figure 3). Respondents who were highly and extremely annoyed by aircraft noise had higher proportion of negative evaluations on soundscape. Respondent’s self-reported annoyance levels were correlated to soundscape ratings, strongest to the evaluation of “artificial-natural” (*r* = 0.417, *p* < 0.001), followed by “unpleasant-pleasant” (*r* = 0.327, *p* < 0.001). Loud (*r* = 0.284, *p* < 0.001) and chaotic (*r* = 0.278, *p* < 0.001) were also associated with the degree of annoyance.

The evaluation of “quiet-loud” was not different by study sites. However, 71% of the respondents in Site II and 64% in Site III rated the environment as pleasant, which was significantly higher than the proportion of 23% in Site II (χ^2^(10) = 53.36, *p* < 0.001). Similarly, Site III with a small water fall in the space also had a higher rating of “predictable” among other sites (χ^2^(10) = 35.80, *p* < 0.001). Site III had the highest proportion of “natural” among other sites (χ^2^(10) = 69.21, *p* < 0.001), possibly due to the prominent sound of birdsong in public spaces.

### 3.3. Effects of community factors

Regression analysis was used to model respondents’ annoyance levels with aircraft noise exposure at their residential area, controlling for community factors (Table 1). The dependent variable – annoyance level – was obtained using a 0-to-10 opinion scale for assessing how much aircraft noise bothers, disturbs or annoys the respondents. Results show that community factors significantly influenced occupants’ annoyance to aircraft noise. Other things being equal, new residents and property owners were more likely to be annoyed by aircraft noise. Respondents who used public space frequently had a higher degree of annoyance.

**TABLE 1:** Regression analysis modeling annoyance level (0–10) using aircraft noise exposure and community factors (*n* = 186, adjusted-*R*^2^ **=** 0.74).

Variables	*B* (95% CI)	*p*-value	*t*
Noise exposure (SPL)	0.16 (0.08, 0.23)	0.000	4.15
Years of living	−0.50 (−0.65, −0.35)	0.000	4.78
Ownership (ref: owned outright)			
- Being bought on mortgage	−0.45 (−0.91, −0.01)	0.048	-1.99
- Rented	−1.00 (−1.55, −0.46)	0.000	-3.65
- Temporarily living	−1.46 (−1.99, −0.93)	0.000	-5.40
Frequency of public space using	1.32 (1.18, 1.46)	0.000	18.71
(Constant)	−7.56 (−12.77, −2.35)	0.005	-2.86

### 3.4. Effects of psychological and demographic factors

To identify vulnerable populations that should be protected from aircraft noise, regression analysis was used to show how residents’ annoyance to aircraft noise was affected by psychological and demographic factors.

Regression model in Table 2 shows that personal noise sensitivity and fear of flight traveling significantly increased the level of annoyance, whilst frequent flight travelers were significantly less annoyed by aircraft noise.

**TABLE 2:** Regression analysis modeling annoyance level (0–10) using aircraft noise exposure and psychological factors (*n* = 186, adjusted-*R*^2^ **=** 0.68).

Variables	*B* (95% CI)	*p*-value	*t*
Noise exposure (SPL)	0.14 (0.05, 0.23)	0.002	3.21
Noise sensitivity	0.58 (0.34, 0.82)	0.000	-6.51
Frequency of travel	−0.64 (−0.88, −0.40)	0.000	-0.530
Fear of flight	1.04 (0.84, 1.24)	0.000	10.12
(Constant)	−6.69 (−12.23, −1.15)	0.018	-2.38

In terms of demographic factors, age and being female increased the level of annoyance (Table 3). Respondents with high household incomes seemed less likely to feel annoyed by the noise. Among all respondents, people with less than high school education were most annoyed be aircraft noise. However, higher education than a bachelor’s degree did not significantly reduce the level of annoyance.

**TABLE 3:** Regression analysis modeling annoyance level (0–10) using aircraft noise exposure and demographic factors (*n* = 186, adjusted-*R*^2^ **=** 0.58).

Variables	*B* (95%CI)	*p*-value	*t*
Noise exposure (SPL)	0.25 (0.15, 0.35)	**0.000**	4.94
Age	0.05 (0.03, 0.07)	**0.000**	5.62
Female	1.36 (0.85, 1.8)	**0.000**	5.27
Household income	−0.29 (−0.57, −0.02)	**0.036**	-2.12
Highest education (ref: no qualification)			
- High school	−0.74 (−1.47, −0.01)	**0.046**	-2.010
- Undergraduate	−1.04 (−1.78, −0.29)	**0.007**	-2.73
- Postgraduate	−0.72 (−1.64, 0.20)	0.125	-1.54
(Constant)	−13.03 (−19.77, −6.29)	**0.000**	-3.81

Statistical significant effects in boldface.

### 3.5. Effects on general health

Respondent’s self-reported health was not directly associated with aircraft noise levels. The study has found that the degree of noise annoyance significantly increased the possibility of poor health status. The effect of annoyance on general health was assessed using logistic regression analysis, modeling the odds of poor health status with noise annoyance and personal factors (Table 4). The dependent variable - poor health status - was binarized from a 5-point scale assessment of general health with poor and fair health classified as 1 (*n* = 74). Respondent’s annoyance to aircraft noise was added into the model as independent variable. Key moderating variables of health, including age, gender and household income were controlled for. Result shows that higher degree of annoyance to aircraft noise was associated with a higher probability of poor health status (OR = 1.36, *p* = 0.017). It should be noted that a significant relationship between noise annoyance and health should not be taken as evidence of a causal pathway from the aircraft noise to health, as the study method did not establish causality between variables, e.g., poor health might cause annoyance, in the reverse direction.

**TABLE 4:** Binary logistic regression showing the association between poor health, annoyance with aircraft noise and covariates (*n* = 187, Nagelkerke-*R^2^* **=** 0.43).

Variables	Odds ratio (95% CI)	*p*-value
Annoyance (0–10)	1.36 (1.06, 1.75)	**0.017**
Age	1.08 (1.05, 1.11)	**0.000**
Female	0.37 (0.14, 1.01)	0.052
Household income	0.71 (0.46, 1.09)	0.122
(Constant)	0.02	**0.000**

Statistical significant effects in boldface.

## 4. Discussion

The study was carried out during the COVID-19 pandemic, when residents might suffer more psychological discomfort than usual and rely on the restoration in community public spaces. Aircraft noise and other sound at the community have profoundly influence resident’s sound perception and well-being. This paper has shown that 88% of the respondents were annoyed by aircraft noise and 50% of them considered their level of annoyance to have deepened during the pandemic. The results might indicate that the negative effects of aircraft noise were enhanced in special times, especially for frequent space users and those residents in poor health status.

Traditional aircraft noise impact studies have mostly focused on dose response curves between absolute aircraft noise exposure and annoyance levels (Miedema and Vos, 1998; Babisch and Kamp, 2009). This study has demonstrated that community soundscape might influence aircraft noise evaluation. Under conditions of similar levels of aircraft noise exposure, Site II and III with natural sounds had lower proportion of highly annoyed respondents and higher proportion of positive ratings (such as pleasant, natural and predictable) than Site I. The findings implied that soundscape features of the community significantly moderated the annoyance to aircraft noise, which might indicate the masking or restorative effects of natural sounds as reported in previous studies (Jeon et al., 2021). It is worth noting that the moderating effect could due to the existence of other visual and audial variables of the public space that might not be controlled for. Nevertheless, this paper can be seen as a further validation of a previous experimental study, which found the effects the natural sounds on moderating the psychophysiological indices of the subjects when exposing to aircraft noise (Qu and Xie, 2022).

The results further approved some previous findings that the variance in people’s annoyance could be better explained when adding non-acoustic factors into the statistical model, which include gender, noise sensitivity, attitudes, and adaptability (Bartels et al., 2018). This paper indicated that the fear of air travel, noise sensitivity, and the frequency of outdoor activity had increased the level of annoyance, which agree well with previous findings (Basner et al., 2017).

This paper provided new empirical findings on the research of aircraft noise and human wellbeing. In previous studies, the effects of aircraft noise on psychological health have not been conclusively established compared to physical illness, but there has been a growing number of studies focusing on subjective health indicators (Lawton and Fujiwara, 2016; Baudin et al., 2018). The effects of aircraft noise annoyance on general health were demonstrated in this study. It is worth noting that the positive association between annoyance and poor health might not due to noise exposure, but related to personal situation, as both variables were examined by respondent’s subjective evaluation.

## 5. Conclusion

The paper has provided empirical support for assessing the impact of fly-over aircraft noise on under-route communities. It demonstrated the effects of aircraft noise on resident’s noise perception and health, controlling for the soundscape, community and personal factors. Results from the field survey showed that resident’s negative perception of aircraft noise could be moderated by soundscape conditions and influenced by noise sensitivity, personal attitude to air travel, education, gender and age factors. Respondent’s annoyance to aircraft noise was also associated with the probability of adverse evaluation on sound environment and poor health status. The findings can inform noise control and community consultation strategies to protect vulnerable populations and improve the overall sound environment quality. The results of the study anticipate the potential to reduce the adverse health impacts of aircraft noise with the creation of community soundscape facilities such as bird-attractive vegetation, small fountains and waterfalls. Future research could use a larger sample of communities to explore the mitigating effects of soundscape facilities on aircraft noise evaluations. Future research directions might also include the possibility of conducting a pre- and post-experiment to estimate the potential causal impact of the soundscape intervention.

## Data availability statement

The raw data supporting the conclusions of this article will be made available by the authors, without undue reservation.

## Ethics statement

The studies involving human participants were reviewed and approved by the Medical Ethics Committee in School. The patients/participants provided their written informed consent to participate in this study.

## Author contributions

FQ developed the research framework, carried out the resident survey and experiments, data analysis, and writing of the manuscript. ZL and WH participated in the questionnaire study, experiments, and statistical analyses. TZ helped with the subject recruitment, statistical data analysis, and reviewed and corrected the manuscript. All authors approved the final version of the manuscript.
