# Supplementary material for: Soundscape and subjective factors affecting residents’ evaluation of aircraft noise in the communities under flight routes
Source: Front Psychol. 2023 Jul 3;14:1197820. doi: 10.3389/fpsyg.2023.1197820 (PMC10350505; doi:10.3389/fpsyg.2023.1197820)
Supplement: Supplementary file 1 [file Data_Sheet_1.PDF]

Environmental Health And Comfort Questionnaire For Residential Areas

Dear Madam/Sir:

We kindly ask you to participate in this survey, in order to investigate the living conditions of the residents and the quality of the community environment, and to help the residents' committee to carry out its work better and create a healthy and comfortable community. The survey will be anonymous and all your information will be kept strictly confidential. It will take 5 minutes of your time, so please answer all the questions carefully and comprehensively according to your own perceptions. Your valuable comments will form an important basis for our research and we look forward to receiving your full answers, thank you!

Q1. In general, would you say your health is...

1☐Poor 2☐Fair 3☐Good 4☐Very good 5☐Excellent

Q2. Taking all things together, on a scale of 0 to 10, how happy would you say you are? Here 0 means you are very unhappy and 10 means you are very happy.

Very unhappy — Very happy

☐	☐	☐	☐	☐	☐	☐	☐	☐	☐	☐
0	1	2	3	4	5	6	7	8	9	10

Q3. Please choose ALL the statement(s) which describe your sleep.

1☐ My sleep is not disturbed at all.	4☐ I occasionally wake up but I soon go back to sleep.
2☐ It's hard for me to fall asleep.	5☐ I often lie awake for a while.
3☐ I sleep less deeply than I would like.	6☐ I have to take sleeping pills to fall asleep.

Q4. Did you experience any of the below during the past week? Please indicate whether you consider it to be caused by wind turbine noise.

	Not at all	Some of the time	All the time
Headache	☐	☐	☐
Nausea	☐	☐	☐
Dizziness	☐	☐	☐
Ear discomfort	☐	☐	☐
Cardiovascular disease	☐	☐	☐
Stress	☐	☐	☐
Tension and edginess	☐	☐	☐
Difficulty in intellectual activities	☐	☐	☐
Other____________(Please specify)	☐	☐	☐

Q5. The following are several things that might exist in people' s living environment. Please state for each thing of the below, whether you notice them and if so, whether you are annoyed by them when you spend time at home.

	Notice? Yes	Notice? No	If you notice, do you find it annoying? 1 (Not at all)	2	3	4	5 (Extremely)
a) Unpleasant odor from outside							
b) Noise from neighbours							
c) Traffic noise							
d) Noise from wind turbines							
e) Bugs, pests or vermin							
f) Vibration of the building							
g) Pollution, grime or dust							
h) Other noise sources_______________(Please specify)							

Q6. Thinking about the last 12 months, when you are at home, how much does noise from wind turbines bother, disturb or annoy you?

1☐Not at all 2☐Slightly 3☐Moderately 4☐Very 5☐Extremely

Q7. Thinking about the last 12 months, what number from 0 to 10 best shows how much you are bothered, disturbed or annoyed by wind turbine noise when you spend time outdoors and indoors at your dwelling?

Not at all — Extremely

0	1	2	3	4	5	6	7	8	9	10
☐	☐	☐	☐	☐	☐	☐	☐	☐	☐	☐

Q8. Thinking about the last 12 months, when you are at home, how loud do you feel the noise of the aircraft?

1☐Not at all 2☐Slightly 3☐Moderately 4☐Very 5☐Extremely

Q9. In terms of environmental noise, how much do you agree or disagree with the following statements?

	1 (Agree strongly)	2	3	4	5	6 (Disagree strongly)
a) I find it hard to relax in a place that's noisy.						
b) I get used to most noises without much difficulty.						

Q10.How often did you travel by air in the past year?

1☐ 0-4 times 2☐ 4-12 times 3☐ More than 12 times

Q11. Do you feel anxious about flying?

1☐Not at all 2☐Slightly 3☐Moderately 4☐Very 5☐Extremely

Q12. Thinking about the last week, how often did you use the public space below your residence?

1☐ Hardly ever used	4☐ Once a day
2☐ Once a few weeks	5☐ Several times a day
3☐ Once a few days	

Q13. Please choose ONE statement which best describes your household's ownership of the accommodation.

1☐Owned outright 2☐being bought on mortgage 3☐Rented 4☐Other

Q14. Thinking about the last week, how often can you see aircraft flying over from where you live?

1☐ 0-5 times 2☐ 5-10 times 3☐ More than 10 times

Q15. How long have you lived at your current address? __________ years

Q16. Your age in years:____________

Q17. Your gender: 1☐Male 2☐Female

Q18. What is your current marital status?

1☐ Single	3☐ Separated / Divorced
2☐ Married / Cohabiting	4☐ Widowed

Q19. What is the highest educational or school qualification have you obtained?

1☐ Junior High School and below	3☐ Undergraduate
2☐ High School	4☐ Postgraduate

Q20. Which one represents the total annual income of your household before any deductions?

1☐30-80k 2☐80-300k 3☐300k-1 million 4☐1 million and above
